# BMPs: Conserved Morphogens in Neural Patterning

**DOI:** 10.1371/journal.pbio.0040346

**Published:** 2006-09-12

**Authors:** Françoise Chanut

During evolution, organisms seem to maintain the function of certain key genes but vary the mechanisms that call these genes into action. For instance, fruit flies and vertebrates share three genes—*vnd*, *ind*, and *msh*—that induce distinct cell fates in the developing nervous system. Both flies and vertebrates express these genes in three adjacent stripes that span the length of their nascent nerve cords. But stripe patterns in fruit flies depend on the nuclear protein Dorsal and in vertebrates on the signaling molecule Hedgehog; both factors are produced in ventral regions of the nerve cord. Since Dorsal and Hedgehog belong to unrelated signaling pathways, vertebrates and fruit flies seem to have independently evolved distinct means of expressing *vnd*, *ind*, and *msh* in the same striped pattern. But in a recent study, Claudia Mieko Mizutani, Ethan Bier, and colleagues demonstrate that both flies and vertebrates similarly rely on a third dorsally produced signal, the bone morphogenetic proteins (BMPs), to paint stripes along their nerve cords. They propose that BMPs are the ancestral patterning signal that the precursors of flies and vertebrates originally shared and that later were reinforced by recruiting either Dorsal or Hedgehog in neural development.

BMPs are extracellular proteins that function early in embryonic development to stem neural differentiation and promote epidermal cell fates. As a result, the nervous system of flies and vertebrates arises only where other extracellular proteins known as Chordin or Sog stave off the influence of BMPs. But in many developmental processes, BMPs act as morphogens: they diffuse away from their source and create patterns in neighboring tissues by regulating gene expression in a dose-dependent manner. As development proceeds, Chordin and Sog subside while the BMPs continue bathing the epidermis. Mizutani thought that the BMPs might also act as morphogens in the developing nerve cord, but that their role would be masked by the more prominent effect of Dorsal or Hedgehog. Thus, to unmask any possible effect, the researchers erased all trace of Dorsal or Hedgehog pattern.

In flies, the Dorsal protein has a graded distribution that peaks at the embryo’s ventral midline and tapers off midway around the embryo’s circumference. High Dorsal concentrations elicit expression of *vnd* in two ventro-lateral stripes (one on each side of the embryo), while intermediate Dorsal doses turn on *ind* at a middle location. The dorsal *msh* stripes lie next to the source of Dpp, the fly BMP, which emanates from the embryo’s dorsal half. By manipulating the genetic control of Dorsal distribution, Mizutani and her colleagues created embryos with a uniform, intermediate level of Dorsal protein, and no Dpp. In these lateralized embryos, all patterning by Dorsal is erased: *ind* is expressed around the whole embryo’s circumference, and *vnd* and *msh* remain silent.

**Figure:**
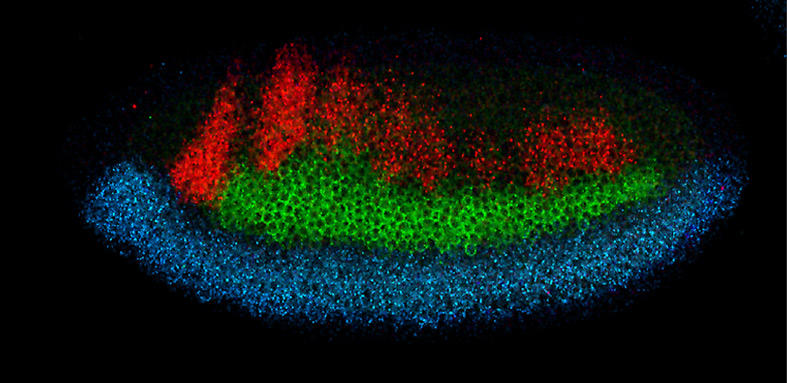
Localized inhibition of BMP signaling in an early *Drosophila* embryo leads to a dorsal shift in the expression domains of neural identity genes. (Image: Claudia Mieko Mizutani)

When the researchers genetically introduced a local source of Dpp in lateralized fly embryos, part of the stripe pattern was recreated: *ind* was repressed locally and made room for *msh* expression close to the Dpp source. This relative pattern of *msh* and *ind* expression is similar to that in normal embryos and indicates that Dpp represses expression of *ind* more effectively than *msh*. While this experiment shows that Dpp can act as a morphogen in the fly nervous system, it doesn’t demonstrate that Dpp is actually required to do the same thing during normal development. To address this question, the researchers created local disruptions of Dpp signaling in normal embryos by expressing Brinker—a nuclear protein that blocks Dpp signaling—in a narrow stripe perpendicular to the *vnd*, *ind*, and *msh* stripes: all three stripes swerved dorsal-ward when they crossed Brinker. This result confirms that Dpp normally patterns the fly’s nervous system by limiting the dorsal expansion of the *vnd*, *ind*, and *msh* stripes.

To test whether BMPs function analogously in patterning of the nervous system in vertebrates, co-authors Henk Roelink and Néva Meyer ventralized fragments of chick neural tubes by soaking them in a Hedgehog solution. The fragments originally expressed only *Nkx2.2*, *vnd*’s vertebrate homolog. But when cultured next to a tissue fragment releasing BMPs, the fragments activated the intermediate and dorsal neural genes *Pax6* and *Msx* in concentric rings, recapitulating the expression pattern seen in normal neural tubes. Hence, in vertebrates as well, BMPs alone can recreate most of the dorso-ventral pattern of neural gene expression. This suggests a conserved role for BMP function during early and late patterning of the neuroectoderm. This unified view in which BMPs repress neural genes in a dose-dependent fashion runs contrary to the prevailing view of vertebrate neural patterning, since BMPs have been proposed to turn on genes in dorsal regions of the neural tube.

A morphogen shared by flies and vertebrates is likely to come from their common ancestor, which was small, according to fossil data. The researchers speculate that a BMP gradient may have been sufficient to pattern the nerve cord of a small organism. But as organisms grew larger, the need arose for additional patterning mechanisms to complement the BMPs, which eventually lost their preeminence in nervous system patterning.

